# SCClone: Accurate Clustering of Tumor Single-Cell DNA Sequencing Data

**DOI:** 10.3389/fgene.2022.823941

**Published:** 2022-01-27

**Authors:** Zhenhua Yu, Fang Du, Lijuan Song

**Affiliations:** ^1^ School of Information Engineering, Ningxia University, Yinchuan, China; ^2^ Collaborative Innovation Center for Ningxia Big Data and Artificial Intelligence Co-founded by Ningxia Municipality and Ministry of Education, Ningxia University, Yinchuan, China

**Keywords:** single-cell sequencing, next-generation sequencing, cancer genome, intra-tumor heterogeneity, clustering

## Abstract

Single-cell DNA sequencing (scDNA-seq) enables high-resolution profiling of genetic diversity among single cells and is especially useful for deciphering the intra-tumor heterogeneity and evolutionary history of tumor. Specific technical issues such as allele dropout, false-positive errors, and doublets make scDNA-seq data incomplete and error-prone, giving rise to a severe challenge of accurately inferring clonal architecture of tumor. To effectively address these issues, we introduce a new computational method called SCClone for reasoning subclones from single nucleotide variation (SNV) data of single cells. Specifically, SCClone leverages a probability mixture model for binary data to cluster single cells into distinct subclones. To accurately decipher underlying clonal composition, a novel model selection scheme based on inter-cluster variance is employed to find the optimal number of subclones. Extensive evaluations on various simulated datasets suggest SCClone has strong robustness against different technical noises in scDNA-seq data and achieves better performance than the state-of-the-art methods in reasoning clonal composition. Further evaluations of SCClone on three real scDNA-seq datasets show that it can effectively find the underlying subclones from severely disturbed data. The SCClone software is freely available at https://github.com/qasimyu/scclone.

## 1 Introduction

Cancer is a dynamic disease driven by accumulation of somatic mutations (Nowell, 1976). The genetic aberrations give cancerous cells a growth advantage over surrounding normal cells to resist apoptosis. With the clonal expansions, distinct subclones presenting genotypic and functional diversity emerge in the tumor (Greaves and Maley, 2012; Swanton, 2012), and their lineage relationship can be depicted in an evolutionary tree. Each branch of the tree forms taxa descended from a common ancestor. As intra-tumor heterogeneity of tumor constitutes one of the critical factors that contribute to therapy resistance, an accurate inference of tumor subclones and their lineage relationship is essential for finding driver genes (Xi et al., 2020) and the assessment of drug resistance and design of personalized treatment.

Next-generation sequencing (NGS) (Metzker, 2010) has shown significant advantages in deciphering the intra-tumor heterogeneity and evolutionary history in tumors. The typical usage of NGS techniques is the sequencing from cells in bulk. The mutation profile obtained from bulk sequencing is a mixed signal that represents an average of thousands or even millions of cells that derive from distinct subclones in the tumor. Therefore, a deconvolution of the mixed signal is required to identify the subclones and recover the clonal lineage. The main challenge lies in the ambiguity that the number of present subclones, their respective prevalence, mutation profiles, and phylogenetic relationships are all undetermined (Navin, 2014). To solve this problem, an abundance of computational approaches has been developed in the last decade to decode clonal composition from bulk-sequencing data (Kuipers et al., 2017; Satas and Raphael, 2017; Yu et al., 2017; Eaton et al., 2018). However, deconvolution-based results suffer from low-resolution indication of clonal architecture due to insufficient coverage of low-prevalence subclones. The detection resolution can be improved by analyzing multiple samples per patient that could either be from spatially distinct regions of tumor (Gerlinger et al., 2014), metastasis, or tumor/relapse pairs (Ding et al., 2012). The snapshots of the tumor at different time points can also be utilized to strengthen the resolution but are usually unavailable.

Single-cell DNA sequencing (scDNA-seq) (Gawad et al., 2016) now provides an unprecedented view of the intra-tumor heterogeneity at single cell resolution. In scDNA-seq techniques, picograms of DNA from isolated single cells are amplified to micrograms of genetic material, producing enough DNA to be sequenced using NGS instruments. Mutation profiles of single cells obtained from scDNA-seq experiments can be exploited to reconstruct the evolutionary tree without the signal deconvolution step as required in bulk sequencing. However, processing scDNA-seq data is usually complicated by several critical issues, such as allele dropout (ADO), false-positive (FP) errors, missing data, and cell doublets (Navin, 2014). ADO can result in false-negative (FN) errors, that is, heterozygous sites are erroneously recorded as homozygous genotypes, and the FN rates reported in previous studies change from 0.1 to 0.43 (Hou et al., 2012; Gawad et al., 2014). FP errors refer to falsely predicting homozygous genotypes to be heterozygous and occur with a higher rate than the somatic mutations (Hou et al., 2012; Xu et al., 2012). Missing sites may result from non-uniform sequencing coverage and ADO events, and the proportion of missing data can exceed 50% in scDNA-seq data (Hou et al., 2012). Cell doublet is another type of noise in scDNA-seq data that derives from unintended capturing of two or more cells when isolating single cells, and the reported doublet rate may reach 10% in current droplet-based techniques (Zafar et al., 2017). These issues usually come together in scDNA-seq data, making it very complicated to get unbiased inference from the data.

So far, an arsenal of computational methods (Jahn et al., 2016; Zafar et al., 2017; El-Kebir, 2018; Chen et al., 2020; Myers et al., 2020; Yu et al., 2021) has been developed to reconstruct tumor phylogeny from single nucleotide variation (SNV) data of single cells. Typically, three popular evolutionary models, that is, the infinite sites model (ISM), the finite site model (FSM), and the Dollo parsimony model, are employed in these methods. The ISM assumption stipulates that each mutation is gained once and will not be lost, and the FSM relaxes the constraint to allow parallel evolution and mutation loss, while the Dollo parsimony model only permits back mutation. For instance, SCITE (Roth et al., 2016) takes ISM assumption and deduces the optimal phylogeny based on the Markov Chain Monte Carlo (MCMC) approach. With finite site assumption, SiFit (Zafar et al., 2017) leverages an MCMC-based approach to infer the cell lineage tree, and the authors further develop SiCloneFit (Zafar et al., 2019) to decipher the clonal evolutionary tree with a doublet model. To model back mutation, SPhyR (El-Kebir, 2018) exploits the Dollo parsimony model to efficiently estimate tumor phylogeny. Similarly, SASC (Ciccolella et al., 2021b) infers loss-supported cancer progression based on simulated annealing. PhISCS-BnB (Sadeqi Azer et al., 2020) delivers perfect phylogeny using a branch and bound algorithm. Recently, GRMT (Yu et al., 2021) is proposed to reconstruct the mutation tree with a generative model. There are some methods that exploit additional data to improve the inference accuracy. For instance, ScisTree (Wu, 2020) incorporates genotype uncertainty information into analysis for better inference of the cell lineage tree. SCARLET (Satas et al., 2020) is a more recently proposed method to infer loss-supported tumor phylogeny refined by copy number profiles.

Inference of subclones constitutes another paradigm for scDNA-seq data analysis. For instance, OncoNEM (Ross and Markowetz, 2016) finds subclones by reasoning the subclonal tree using a heuristic search algorithm and fine-tunes the tree with unobserved subclones. SCG (Roth et al., 2016) uses a hierarchical Bayesian model to cluster single cells into distinct subclones. RobustClone (Chen et al., 2020) is proposed to efficiently recover subclonal composition with no explicit restriction on the evolutionary model. Furthermore, BnpC (Borgsmüller et al., 2020) adopts a non-parametric approach to cluster cells into subclones. Another method called celluloid (Ciccolella et al., 2021a) intends to reduce scDNA-seq data size for efficient reconstruction of tumor phylogeny *via* mutation clustering before tree inference. Despite the acceptably good performance of existing clustering methods, their applications may encounter specific limitations. The heuristic search and MCMC-based methods are shown to suffer from high computational complexity; therefore, they cannot scale well to large scDNA-seq datasets. In addition, the performance of the existing methods on severely disturbed scDNA-seq data, such as datasets complicated by a high FN rate, is not yet fully investigated, and they may suffer from heavy performance degradation on such hard cases. Therefore, clustering of binary mutation data is still a challenging task, and methods for accurate and efficient clustering of scDNA-seq data are still highly needed for deciphering subclones of tumor.

In this study, we introduce SCClone, a novel method for inferring intra-tumor heterogeneity from scDNA-seq data by addressing aforementioned critical issues. SCClone clusters single cells into distinct subclones by formulating the input genotype matrix (GTM) under a probability mixture model for binary data. Unlike the existing search-based methods, SCClone directly learns subclonal mutational profiles and FP and FN rates from input data *via* an expectation-maximization (EM) algorithm and therefore converges faster than the MCMC-based methods. In addition, a novel model selection approach based on inter-cluster variance is proposed to accurately decipher underlying clonal composition. Comprehensive evaluations on various simulated datasets demonstrate that SCClone performs better than the state-of-the-art methods in multiple evaluation metrics. We also validate the effectiveness of SCClone on three real scDNA-seq datasets.

## 2 Materials and Methods

**FIGURE 1:**
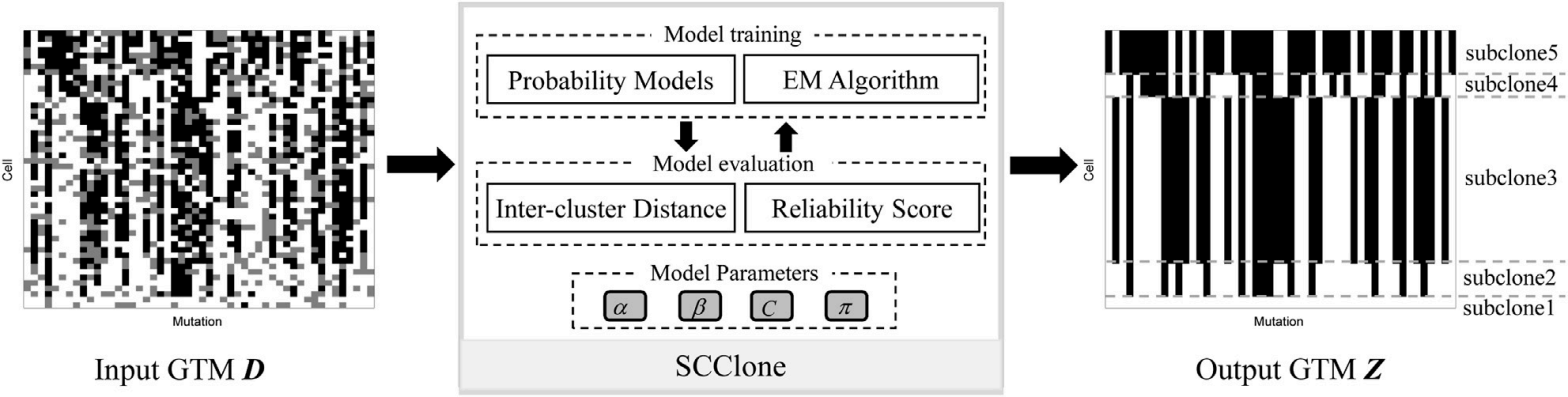
Schematic overview of the SCClone framework. SCClone takes noisy genotype matrix *D* inferred from scDNA-seq data as input and infers the number of subclones as well as the genotypes of each subclone. Each element of *D*_*ij*_ denotes the state (presence, absence, and unobserved) of the *j*th mutation in the *i*th cell, and the different states are marked by black, white, and gray, respectively.

The input to SCClone is a binary *N* × *M* GTM *D* that depicts the observed genotypes of *N* cells at *M* genomic loci. The outputs of SCClone include 1) underlying subclones from which the observed mutation data derive and 2) estimated proportions of false negatives and false positives in the mutation data. A schematic illustration of SCClone workflow is given in Figure 1. SCClone employs an EM-based clustering model to decipher the clonal composition of the tumor and the error rates.

### 2.1 Probability Models for Formulating Mutation Data

Given the observed *N* × *M* GTM *D*, we denote the ground truth GTM as Z* and assume each cell derives from one of the *K* cell populations. Cells from the same population constitute a separate cluster. The mutation states of the *k*th cluster are denoted by a vector *C*_*k*_ of length *M*, where each element *C*_*kj*_ represents presence (*C*_*kj*_ = 1) or absence (*C*_*kj*_ = 0) of mutation *j* in population *k*. Due to allele dropout and false-positive issues, the observed data *D* are often confounded by FN and FP calls. The conditional probability of the *i*th cell is formulated by the following:

$$p\left(D_{i}|C_{k}\right)=\overset{M}{\underset{j=1}{\prod }}p\left(D_{ij}|C_{kj}\right)$$ (1)

with *p* (*D*_*ij*_|*C*_*kj*_) defined as follows:

$$p\left(D_{ij}|C_{ij}\right)=\left(\begin{array}{cc} p\left(0|0\right) & p\left(1|0\right)\\ p\left(0|1\right) & p\left(1|1\right) \end{array}\right)=\left(\begin{array}{cc} 1-\alpha & \alpha \\ \beta & 1-\beta \end{array}\right)$$ (2)

where *α* and *β* indicate the false-positive rate (FPR) and the false-negative rate (FNR), respectively. We rewrite formula (2) to the following form for computational convenience:

$$\begin{array}{cc} p\left(D_{ij}|C_{kj}\right)= & {\left({\left(1-\beta \right)}^{D_{ij}}\beta^{1-D_{ij}}\right)}^{C_{kj}}\\ & {\left({\left(1-\alpha \right)}^{1-D_{ij}}\alpha^{D_{ij}}\right)}^{1-C_{kj}} \end{array}$$ (3)

Suppose the proportion of the *k*th cluster is *π*_*k*_, then the log-likelihood of observed mutation data can be expressed by the following:

$$l\left(C,\pi ,\alpha ,\beta \right)=\overset{N}{\underset{i=1}{\sum }}\log\left(\overset{K}{\underset{k=1}{\sum }}\pi_{k}p\left(D_{i}|C_{k}\right)\right)$$ (4)

We aim to find the maximum likelihood estimation of the model parameters *θ* = (*C*, *π*, *α*, *β*), that is, $\theta^{*}=\underset{\theta }{\arg\max}l\left(\theta \right)$ .

### 2.2 EM Algorithm for Parameter Estimations

We employ an EM algorithm to infer the model parameters. In the E-step, the posterior probability that the *i*th cell belongs to the *k*th cluster is calculated as follows:

$$\gamma_{ik}^{\left(n\right)}=\frac{\pi_{k}^{\left(n-1\right)}p\left(D_{i}|C_{k}^{\left(n-1\right)}\right)}{\sum_{j=1}^{K}\pi_{j}^{\left(n-1\right)}p\left(D_{i}|C_{j}^{\left(n-1\right)}\right)}$$ (5)

based on the current parameters *θ* ^(*n*−1)^. The objective function to maximize in the *n*th iteration of the M-step is the expected partial log-likelihood:

$$J^{\left(n\right)}=\overset{N}{\underset{i=1}{\sum }}\overset{K}{\underset{k=1}{\sum }}\gamma_{ik}^{\left(n\right)}\left(\log\left(p\left(D_{i}|C_{k}\right)\right)+\log\left(\pi_{k}\right)\right)$$ (6)

The value of $C_{kj}^{\left(n\right)}$ can be inferred as $C_{kj}^{\left(n\right)}=\underset{s}{\arg\max}J^{\left(n\right)}\left(C_{kj}=s\right)$ . The parameter *π* is updated under the constraint $\sum_{k=1}^{K}\pi_{k}=1$ . By employing the Lagrange multiplier method, we get the updating formula for *π*_*k*_ as follows:

$$\pi_{k}^{\left(n\right)}=\frac{\sum_{i=1}^{N}\gamma_{ik}^{\left(n\right)}}{N}$$ (7)

By maximizing the objective function with respect to *β*, we derive the rule to update *β*:

$$\beta^{\left(n\right)}=\frac{\sum_{i=1}^{N}\sum_{k=1}^{K}\gamma_{ik}^{\left(n\right)}\sum_{j=1}^{M}C_{kj}^{\left(n\right)}\left(1-D_{ij}\right)}{\sum_{i=1}^{N}\sum_{k=1}^{K}\gamma_{ik}^{\left(n\right)}\sum_{j=1}^{M}C_{kj}^{\left(n\right)}}$$ (8)

The parameter *α* is usually available for scDNA-seq experiments but can also be updated by the following:

$$\alpha^{\left(n\right)}=\frac{\sum_{i=1}^{N}\sum_{k=1}^{K}\gamma_{ik}^{\left(n\right)}\sum_{j=1}^{M}\left(1-C_{kj}^{\left(n\right)}\right)D_{ij}}{\sum_{i=1}^{N}\sum_{k=1}^{K}\gamma_{ik}^{\left(n\right)}\sum_{j=1}^{M}\left(1-C_{kj}^{\left(n\right)}\right)}$$ (9)

The model parameters are iteratively updated until the EM algorithm converges, and the optimal solution is denoted by θ* = (C*, π*, α*, β*). Each cell is then assigned to the cluster associated with the highest posterior probability, and the predicted GTM is denoted as *Z*.

### 2.3 Initialization of Model Parameters

The final solution found by the EM algorithm may heavily depend on the initial values of model parameters; therefore, appropriate configurations of *θ* ^(0)^ = (*C* ^(0)^, *π* ^(0)^, *α* ^(0)^, *β* ^(0)^) are critical to find the optimal solution. Specifically, we adopt a uniform distribution for *π* ^(0)^, set *α* ^(0)^ to 0.01, and perform grid search on *β* ^(0)^. If *α* and *β* are specified by users, their values will not be updated. *C* ^(0)^ is specified *via* random sampling from input mutation data.

### 2.4 Determination of the Best Number of Clusters

To find the best number of clusters, that is, the value of *K*, we introduce a score metric based on inter-cluster variance to evaluate models with different *K* values. The inter-cluster distance measures how well the cells from distinct subclones are separated. Suppose θ* = (C*, π*, α*, β*) represent the inferred optimal parameters for a given *K* and *V*_*k*_ denotes the set of cells predicted to be from the *k*th cluster, we first calculate the expected inter-cluster distance *d* (*i*, *k*) for each pair of clusters (*i*, *k*) as follows:

$$\begin{array}{cc} d\left(i,k\right)= & p_{00}\overset{M}{\underset{j=1}{\sum }}\left(1-C_{ij}^{*}\right)\left(1-C_{kj}^{*}\right)+p_{01}\overset{M}{\underset{j=1}{\sum }}\left(1-C_{ij}^{*}\right)C_{kj}^{*}\\ & +p_{10}\overset{M}{\underset{j=1}{\sum }}C_{ij}^{*}\left(1-C_{kj}^{*}\right)+p_{11}\overset{M}{\underset{j=1}{\sum }}C_{ij}^{*}C_{kj}^{*} \end{array}$$ (10)

where *p*_*st*_ is the conditional probability that two cells *c*_1_ ∈ *V*_*i*_ and *c*_2_ ∈ *V*_*k*_ have different observed states (include missing entries) at a genomic locus given that the mutation states of the *i*th and *k*th clusters at the locus are *s* and *t*, respectively. The values of *p*_*st*_ can be empirically estimated as follows:

$$p_{00}=2\alpha^{*}\left(1-\alpha^{*}\right){\left(1-\eta \right)}^{2}+2\eta \left(1-\eta \right)$$ (11)

$$p_{01}=\left(\left(1-\alpha^{*}\right)\left(1-\beta^{*}\right)+\alpha^{*}\beta^{*}\right){\left(1-\eta \right)}^{2}+2\eta \left(1-\eta \right)$$ (12)

$$p_{11}=2\beta^{*}\left(1-\beta^{*}\right){\left(1-\eta \right)}^{2}+2\eta \left(1-\eta \right)$$ (13)

where *η* is the proportion of missing entries in the input GTM *D* and *p*_10_ = *p*_01_. The distances of all pairs of cells in clusters *i* and *k* are calculated and the mean distance $\hat{d}\left(i,k\right)$ is used to define the inter-cluster score:

$$s\left(i,k\right)=\exp\left(-{\left(d\left(i,k\right)-\hat{d}\left(i,k\right)\right)}^{2}\right)$$ (14)

The larger the value of *s* (*i*, *k*), the higher the probability that clusters *i* and *k* are correctly separated. We then calculate the mean inter-cluster score for all unordered pairs of clusters to evaluate the whole model with *K* clusters:

$$s\left(K\right)=\frac{2}{K\left(K-1\right)}\overset{K-1}{\underset{i=1}{\sum }}\overset{K}{\underset{k=i+1}{\sum }}s\left(i,k\right)$$ (15)

In SCClone, we start with the assumption of tumor homogeneity (*K* = 1), then iteratively increase the number of clusters (*K* = *K*+1) until the maximum value of the score has not changed for more than *κ* (set to 10 by default) times.

### 2.5 Performance Evaluation

The performance of SCClone is compared to three state-of-the-art methods, that is, SCG (Roth et al., 2016), RobustClone (Chen et al., 2020), and BnpC (Borgsmüller et al., 2020), based on several performance metrics adopted in previous studies (Borgsmüller et al., 2020; Chen et al., 2020). To evaluate the clustering accuracy, we calculate the V-Measure (Rosenberg and Hirschberg, 2007) to quantify how well the cells are correctly clustered. In addition, we assess the genotyping accuracy by comparing the predicted GTM *Z* to the ground truth GTM Z* and adopt three metrics for evaluation: accuracy, sensitivity, and specificity. The accuracy is the fraction of correctly called entries in *Z* when compared to Z*, sensitivity is the proportion of correctly identified 1-entries among all 1-entries in Z*, and specificity is calculated as the proportion of correctly called 0-entries among all 0-entries in Z*. All performance metrics are calculated with doublet cells excluded.

The parameter configurations to run each method are as follows: 1) for SCG, the maximum number of clusters is set to $\frac{N}{4}$ , the maximum number of iterations is set to 1 × 10^9^, and the gamma prior is configured as “[9.99, 0.01, 1.0e-15] [2.5, 7.5, 1.0e-15] [1.0e-15, 1.0e-15, 1]”; 2) for running RobustClone, default parameters are used; 3) for BnpC, we set the runtime to $\frac{N}{50}$ minutes to make the model convergent, as suggested by previously reported results (Borgsmüller et al., 2020); and 4) for SCClone, we use default parameters.

### 2.6 Datasets

To make comprehensive comparison of the performance between the investigated methods, we build seven simulated datasets (denoted by D1–D7) under various controlling factors represented by F=(N,M,K,α,β,η,ρ) by following the simulation process introduced in the study by Ross and Markowetz (2016). Here, *ρ* denotes the doublet rate. To simulate mutation data, a subclonal lineage tree is first generated and cells are then assigned to the subclones. The subclonal tree is initialized to only contain two nodes, of which one denotes root, and the remaining subclones are iteratively attached to non-root nodes with uniform probability. Given the simulated subclonal tree, mutations are uniformly assigned to the edges of the tree, and the cells are iteratively assigned to the subclones with the probability of choosing a subclone proportional to the size of the subclone, which enables generation of differently sized subclones. The true genotypes of each cell can be deduced from the subclonal tree by visiting the path from the root to the attachment point of the cell. Finally, the observed GTM is derived from the true GTM by introducing different noises.

Unless indicated otherwise, the default values of the technical factors are set to (*α* = 0.01, *β* = 0.2, *η* = 0.2, *ρ* = 0.1), and each dataset is constructed by changing at least one of the factors. The simulation details of the datasets are as follows: D1 and D2 are formed by 500 × 200 GTMs deriving from *K* = 10 subclones, *β* takes value from {0.2, 0.3, 0.4, 0.5} for D1, and *η* takes value from {0.2, 0.3, 0.4, 0.5} for D2; D3 contains 1,000 × 500 GTMs with *β* = 0.8 and *K* = 10; D4 is constituted by 1,000 × 500 GTMs with the number of subclones *K* sampled from {20, 30, 40, 50} and *β* = 0.3; D5 consists of GTMs with changing number of cells *N* ∈ {500, 1,000, 1,500, 2,000}, *M* = 200, *K* = 15 and *β* = 0.3; D6 is a small-sized dataset and consists of 200 × 50 GTMs with *K* = 5; D7 is produced with *N* = 100, *M* = 100, *α* ∈ (0.01, 0.1), *β* ∈ (0.05, 0.4) and *K* = 5. For each value of the changing factor in D1–D6, 10 replicates are simulated, and D7 contains 50 GTMs. The indexes of doublet cells are also recorded. In addition, we further evaluate SCClone on three real datasets to demonstrate its effectiveness in handling scDNA-seq data.

## 3 Results

### 3.1 Comprehensive Evaluation of SCClone on Simulated Data

#### 3.1.1 SCClone Shows High Robustness Against Different Noises in scDNA-seq Data

**FIGURE 2:**
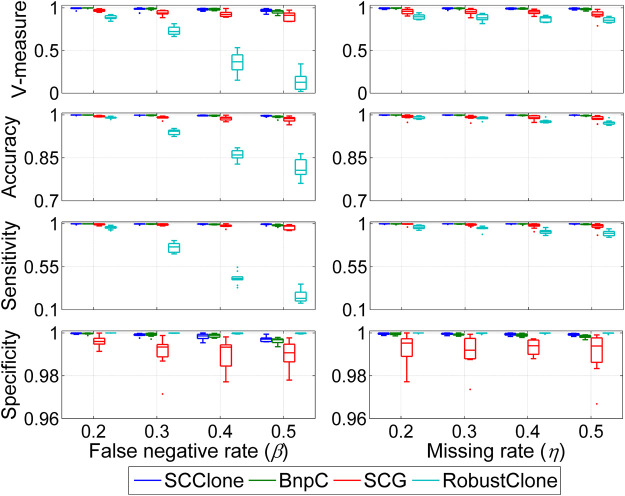
Performance evaluation results on the simulated datasets D1 and D2. The dataset D1 consists of 500 × 200 genotype matrices with the false negative rate changing from 0.2 to 0.5, and dataset D2 is constituted by 500 × 200 genotype matrices with the missing rate ranging from 0.2 to 0.5. Four performance metrics including V-measure, accuracy, sensitivity, and specificity are measured to examine the effects of false-negative errors and missing entries on inference accuracy.

We first evaluate the robustness of different methods against two types of noises including FN errors and missing entries (MEs) on datasets D1 and D2, and the results are shown in Figure 2. The simulated FNR *β* changes from 0.2 to 0.5, and missing rate *η* ranges from 0.2 to 0.5.

When investigating the effects of false-negative errors on inference accuracy, we find SCG can effectively correct FN errors and recover the underlying GTMs across different *β* values, and BnpC shows generally better results than SCG. For instance, the mean V-measure and accuracy of SCG at *β* = 0.5 are as high as 0.902 and 0.984, respectively, and the corresponding metric values of BnpC are 0.954 and 0.993. We can also observe that RobustClone achieves comparable performance to SCG and BnpC when *β* = 0.2, but suffers from degraded accuracy at larger *β* values. For instance, the mean sensitivity of RobustClone decreases from 0.960 at *β* = 0.2 to 0.238 at *β* = 0.5, and the corresponding V-measure decreases by a large margin from 0.889 to 0.139. RobustClone first recovers the genotype matrix without exploiting subclonal information and then clusters the cells based on the inferred genotypes, which may result in suboptimal solutions. SCClone exhibits good robustness against FN errors, and delivers high consistency between the recovered and ground truth GTMs across different *β* values. It reaches 0.967 mean V-measure in clustering cells as well as 0.997 mean accuracy in rebuilding the GTM even at *β* = 0.5.

Besides its better performance in correcting false-negative errors, SCClone also has advantage in dealing with incomplete scDNA-seq data with a high missing rate. As shown in Figure 2, SCClone is the most effective method in extrapolating the MEs and significantly attenuates the effects of MEs on GTM recovery and subclone inference. For instance, the mean V-measure and accuracy of SCClone at *η* = 0.5 are as high as 0.990 and 0.999, and the corresponding metrics of SCG, BnpC, and RobustClone are (0.918, 0.986), (0.984, 0.997), and (0.856, 0.973), respectively.

**FIGURE 3:**
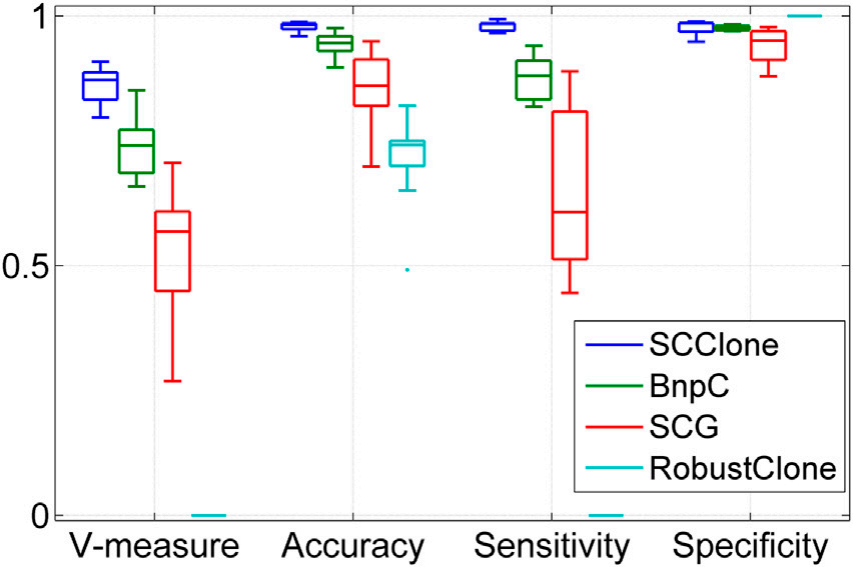
Performance evaluation results on the simulated dataset D3. The dataset consists of 1,000 × 500 genotype matrices with the false negative rate being as high as 0.8.

To verify if the superior performance of SCClone generalizes to more complex datasets, we evaluate SCClone on dataset D3 where the FNR is set to 0.8. The results in Figure 3 suggest SCClone still obtains better results than the state-of-the-art methods and delivers 0.862 mean V-measure as well as 0.979 mean accuracy. BnpC outperforms other existing methods, and RobustClone suffers from severely degraded performance on this dataset. Taken together, these results demonstrate our method has high robustness to FN errors and MEs in scDNA-seq data and gains advantages over the existing methods in accurately recovering the GTM and clustering cells.

#### 3.1.2 SCClone Performs Well in Detecting Subclones

**FIGURE 4:**
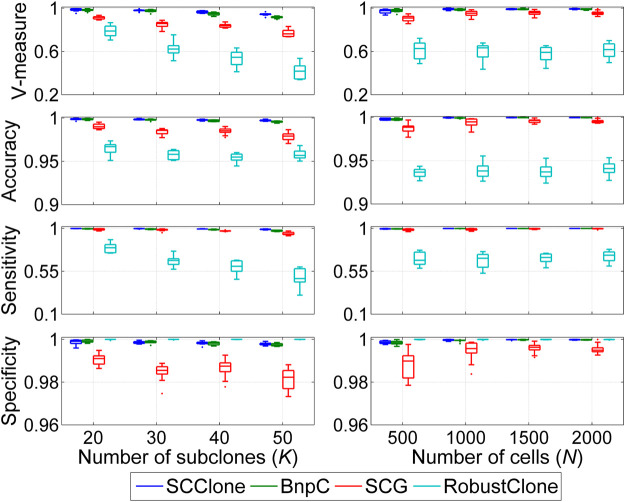
Performance evaluation results on the simulated datasets D4 and D5. The dataset D4 consists of 1,000 × 500 genotype matrices with the number of subclones changing from 20 to 50, and dataset D5 is constituted by genotype matrices with the number of cells ranging from 500 to 2,000.

We further examine the performance of SCClone in reasoning clonal composition with complex lineage structure on dataset D4. The evaluation is conducted on simulated data where the number of subclones *K* changes from 20 to 50, and the results are depicted in Figure 4. BnpC performs better than SCG and RobustClone and yields highly consistent results with the ground truth across different test conditions. Similar to the results on datasets D1–D3, RobustClone has the highest specificity but miss-classifies a large proportion of 1-entries as 0 and delivers less accurate clustering results. For instance, the mean V-measure of RobustClone decreases from 0.786 at *K* = 20 to 0.414 at *K* = 50, while the corresponding values of SCG and BnpC are (0.914, 0.770) and (0.981, 0.915), respectively. Our method exhibits high robustness against the change in clonal structures and achieves good performance at different *K* values.

**FIGURE 5:**
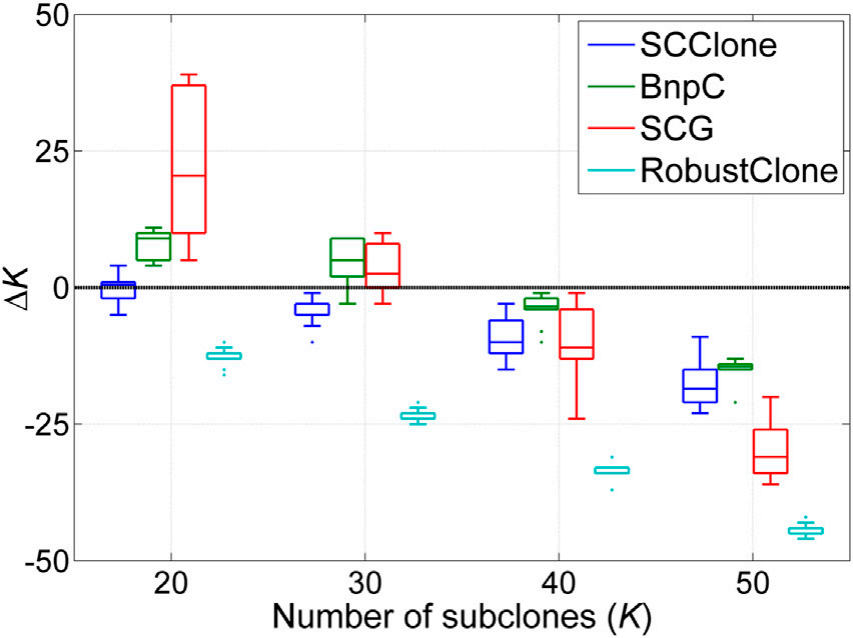
Number of subclones estimated by SCClone, BnpC, SCG, and RobustClone on the simulated dataset D4. The simulated number of subclones changes from 20 to 50. Δ*K* denotes the difference between predicted and expected number of subclones.

We also assess the accuracy of SCClone in inferring the number of subclones and make a comparison with other methods. The results in Figure 5 indicate all methods underestimate the number of subclones on GTMs with *K* ≥ 40, and SCClone performs acceptably well on GTMs with *K* < 40. Although BnpC identifies more subclones than SCClone on complex GTMs, it tends to deliver false-positive calls of subclones since SCClone yields higher clustering accuracy than BnpC as demonstrated in Figure 4. SCClone does not explicitly consider doublet cells when modeling the mutation data, which may be a considerable factor that results in the degraded clustering accuracy of SCClone on the complex GTMs.

#### 3.1.3 SCClone Performs Well on Different-Sized scDNA-seq Datasets

**FIGURE 6:**
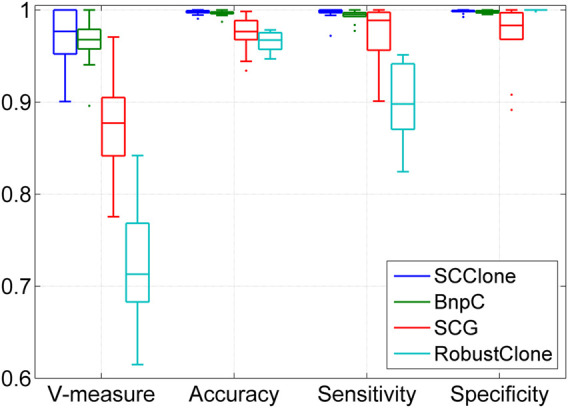
Performance evaluation results on the simulated dataset D6. The dataset consists of 200 × 50 genotype matrices with five subclones.

Besides the superior performance on medium-sized datasets, we proceed to evaluate the scalability of SCClone on large scDNA-seq datasets. To achieve this, up to 2000 cells are simulated to investigate the effect of number of cells on inference accuracy, and the comparison results on dataset D5 are presented in Figure 4. With more cells exploited into the analysis, all methods yield improved results in revealing the clonal composition. BnpC performs better than SCG and RobustClone in recovering the GTM and achieves as high as 0.999 mean accuracy when the number of cells *N* = 2,000. By comparison, SCClone also gives good results and has high accuracy ( > 0.999) when *N* > 500. We also analyze the runtime efficiency of the investigated methods on this dataset. As RobustClone employs a model-free framework to infer clonal composition, it has higher efficiency than other methods. For instance, the mean elapsed time of SCClone, BnpC, SCG, and RobustClone on the GTMs with 2000 cells are 18, 40, 14, and 0.2 min, respectively. SCClone shows comparable computational efficiency to SCG. We further assess the performance of SCClone on small dataset D6 consisting of 200 × 50 GTMs. The results in Figure 6 suggest SCClone is able to accurately cluster single cells and infer the subclonal genotypes. The mean accuracy of SCClone is 0.971 for clustering and 0.998 for genotyping, and the corresponding metrics of BnpC, SCG, and RobustClone are (0.965, 0.996), (0.875, 0.973), and (0.72, 0.965), respectively. Taken together, the evaluation results suggest our method performs well on different-sized scDNA-seq datasets.

#### 3.1.4 SCClone Can Accurately Estimate the Error Rates

**FIGURE 7:**
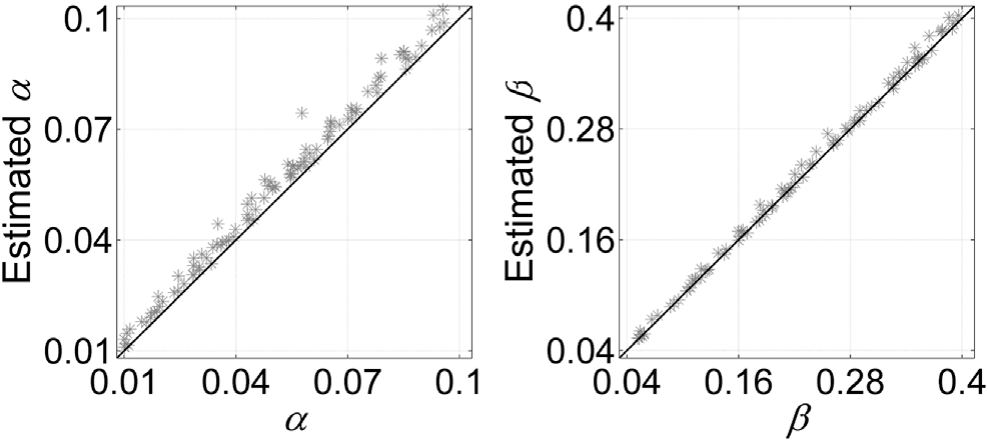
Error rate estimation results of SCClone on the simulated dataset D7. The simulated FPR *α* changes from 0.01 to 0.1, and FNR *β* changes from 0.05 to 0.4.

We also examine the ability of SCClone in estimating the error rates in scDNA-seq data. Evaluations are conducted on simulated dataset D7 with *α* changing from 0.01 to 0.1 and *β* being in range (0.05, 0.4). The results in Figure 7 indicate our method is very effective in accurately estimating the error rates. The predicted values of *α* are highly correlated with the ground truth (coefficient = 0.996). It is also observed that the inferred *α* is generally larger than the simulated value due to doublet cells. Since a doublet event causes a homozygous locus to be recorded as heterozygous provided that any of the cells constituting the doublet mutates at that locus, the doublets inevitably result in the elevated FPR. In addition, SCClone accurately estimates the FNR with highly significant correlation with the true value (coefficient = 0.999), and the estimation of *β* is less affected by doublets. The results demonstrate our method can automatically and accurately estimate both the FPR and FNR from the data.

### 3.2 Evaluation of SCClone on Metastatic Colorectal Cancer Dataset

We use SCClone to infer subclones of metastatic colorectal cancer patient CRC1 (Leung et al., 2017). This dataset consists of 178 single cells isolated from primary and metastatic tumor tissues. Genotype calling finds 16 SNVs among all cells, yielding a 178 × 16 mutation matrix with binary entries.

**FIGURE 8:**
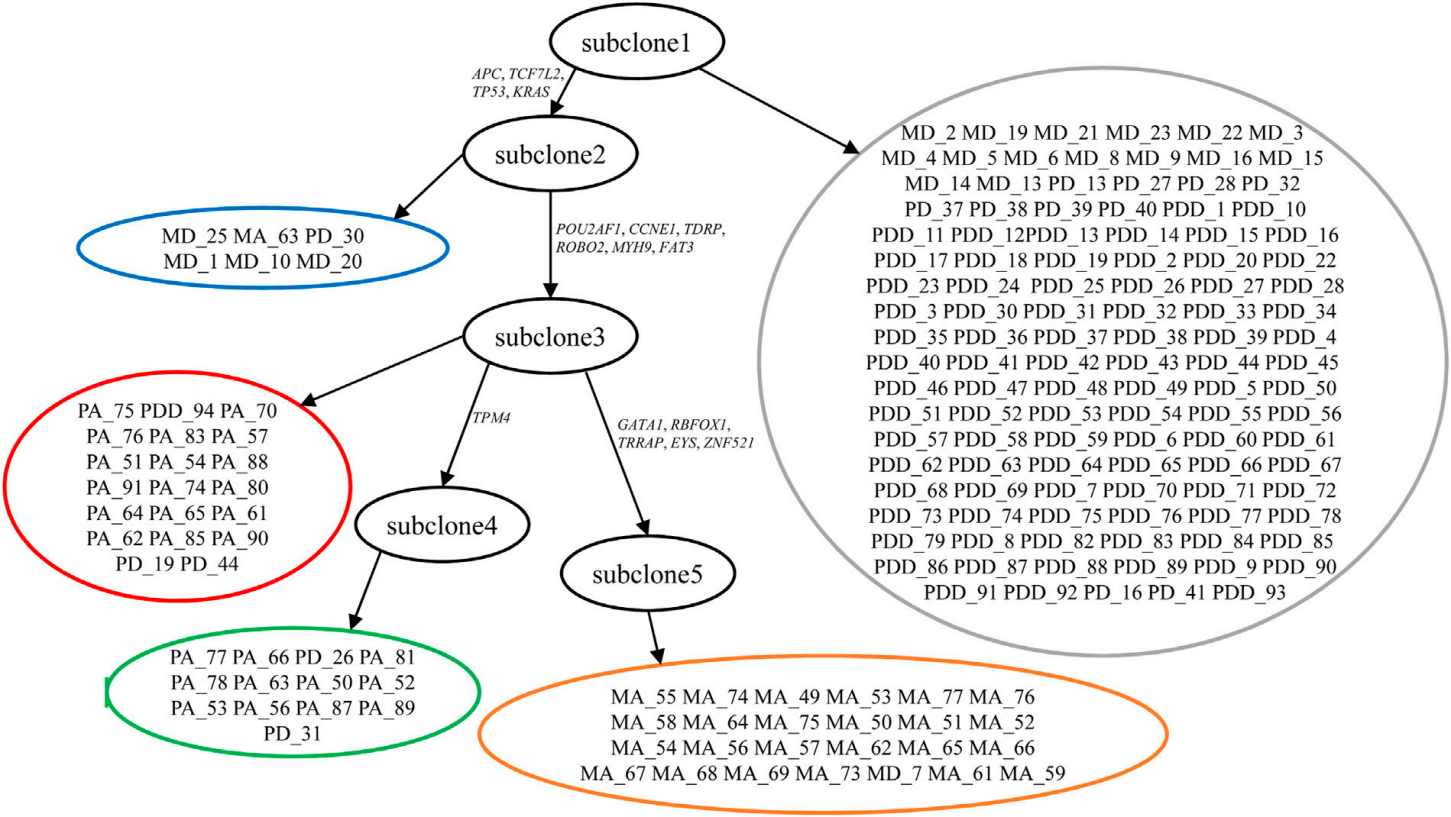
Subclones and their lineage relationship inferred from metastatic colorectal cancer dataset. SCClone identifies five subclones. subclone1 represents normal population without mutations, subclone2 consists of mutated diploid cells, subclone3 and subclone4 are constituted by primary aneuploid cells, and subclone5 represents metastatic aneuploid cells. The estimated FPR *α* and FNR *β* are 0.96 and 14.46%, respectively.

By automatically learning the error rates, SCClone finds five subclones (subclone1 ∼subclone5) in this tumor, and the clonal lineage relationship constructed by the minimum spanning tree (MST) based on genotypes is shown in Figure 8. The error rates are estimated as *α* = 0.96% and *β* = 14.46%. The root of the clonal tree represents diploid cells (marked by gray) without mutations and mainly contains primary diploid cells. Mutations in *APC*, *TCF7L2*, and *TP53* tumor suppressor genes and *KRAS* oncogene result in the emergence of subclone2 (marked by blue). *TCF7L2* is reported to be frequently mutated in CRC and acts as an invasion suppressor (Wenzel et al., 2020). Subclone3 is derived from subclone2 through mutation gains in genes like *POU2AF1*, *CCNE1*, *ROBO2*, and *MYH9*. *CCNE1* is an oncogene that has frequently been amplified in malignancies (Pils et al., 2014), and *MYH9* is considered to promote growth and metastasis in CRC (Wang et al., 2019). This subclone consists mostly of primary aneuploid cells (marked by red). One branch from subclone3 yields a set of primary aneuploid cells (marked by green) that constitute subclone4 through gain of mutation in *TPM4* tumor suppressor gene. Another branch derived from subclone3 is characterized by the mutations in *GATA1*, *RBFOX1*, *TRRAP*, *EYS*, and *ZNF521*. It is noted that *GATA1* is reported as an important gene to promote CRC migration (Yu et al., 2019), and *RBFOX1* deletion occurs with high prevalence in CRC patients (Sengupta et al., 2013). This metastatic clade represents the subclone5 formed by metastatic aneuploid cells (marked by orange).

We compare the subclones inferred by SCClone to the results of SCG, RobustClone, and BnpC. SCG classifies the cells into three clusters including the normal population, a subclone mainly constituted by primary aneuploid cells, and another subclone that represents the metastatic clade. Compared to the results of SCClone, SCG groups all the primary aneuploid cells with and without mutation in *TPM4* into the same cluster and predicts the primary diploid cells to be normal or aneuploid. We also obtain the results of RobustClone on this dataset, and it clusters the cells into one subclone encompassing mutated cells and the normal population without mutations. RobustClone maps all the primary and metastatic aneuploid cells into the same cluster and predicts the mutations in genes like *GATA1*, *RBFOX1*, and *TPM4* as false positives, which is inconsistent with the previously reported results (Zafar et al., 2017). Compared to SCClone, BnpC delivers an over-segmented result on this dataset by dividing the cells into 16 clusters.

### 3.3 Evaluation of SCClone on High Grade Serous Ovarian Cancer Dataset

**FIGURE 9:**
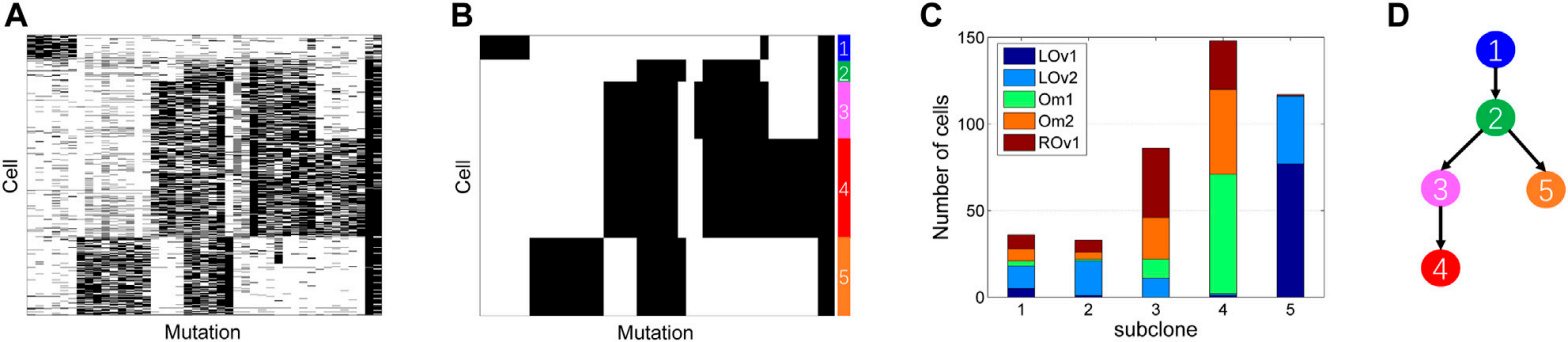
Subclones inferred from high grade serous ovarian cancer dataset. SCClone identifies five subclones and estimates the error rates as *α* = 2.26% and *β* = 31.61%. **(A)** The observed 420 × 43 genotype matrix. **(B)** The recovered genotype matrix and inferred subclones. **(C)** Distribution of the cells among subclones. **(D)** Constructed lineage relationship of subclones by building the minimum spanning tree.

The high grade serous ovarian cancer (HGSOC) dataset contains the mutation states of 420 single cells on 43 genomic loci. The genotype matrix encompasses 10.7% missing entries as shown in Figure 9A. The cells are collected from the left ovary (LOv), right ovary (ROv), and omentum (Om). We apply SCClone on this dataset to infer clonal composition. It finds five subclones (labeled by the numbers 1∼5 as depicted in Figure 9B) and simultaneously estimates the error rates as *α* = 2.26% and *β* = 31.61%. The number of cells assigned to each subclone are 36, 33, 86, 148, and 117, respectively. It is observed that the distribution of the cells is highly different across distinct subclones (Figure 9C). For instance, subclone4 is mainly formed by the ROv and Om cells, while subclone5 consists of the LOv cells. In addition, 91.67% of the LOv1 cells are assigned to subclone5, and 82.14% of the Om1 cells belong to subclone4. These findings are highly in accordance with the reported results in SCG and BnpC. We further analyze the lineage relationship between the subclones by constructing MST (Figure 9D), and get the same topology of the lineage tree as delivered by RobustClone. These results suggest our method is effective in handling medium-sized real scDNA-seq data.

### 3.4 Evaluation of SCClone on IDH-Mutant Gliomas Dataset

**FIGURE 10:**
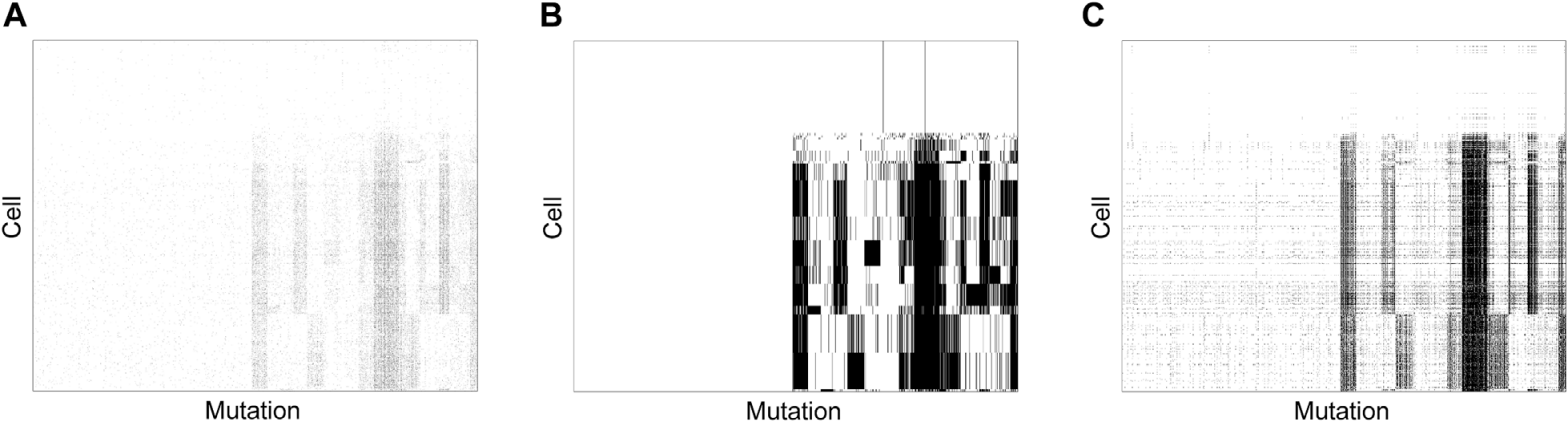
Subclones inferred from IDH-mutant gliomas dataset. SCClone identifies 18 subclones and estimates the error rates as *α* = 0.64% and *β* = 82.38%. **(A)** The observed 926 × 1,392 genotype matrix. **(B)** The recovered genotype matrix and inferred subclones by SCClone. **(C)** The recovered genotype matrix and inferred subclones by BnpC.

To assess if the good performance of SCClone observed on simulated data generalizes to real large scDNA-seq datasets, we further apply SCClone on an IDH-mutant gliomas dataset (Venteicher et al., 2017; Ciccolella et al., 2021a) formed by 926 cells and 1,392 mutations. This dataset consists of a highly sparse GTM with ∼96.8% entries being zero (Figure 10A).

SCClone clusters the cells into 18 subclones (Figure 10B), and predicts 686 mutations to be false positives in any of the subclones. Further analysis suggests 200 of these mutations are singletons (each mutation is only present in a single cell), and our method is able to automatically identify false positives in mutation data without a preprocessing step to filter possible singletons. The estimated FPR and FNR are *α* = 0.64% and *β* = 82.38%, respectively, and such a high FNR may be associated with severe allele dropout events. In addition, distinct mutation patterns are observed among the subclones, and several shared blocks of mutations appear in major subclones. BnpC infers 33 subclones on this dataset (Figure 10C) and predicts the error rates to similar values (*α* = 1.13% and *β* = 76.7%). Compared to SCClone, BnpC delivers over-segmented results for single cells, which implies BnpC is more sensitive to subtle changes between the cells within the same subclone and therefore classifies the cells into different clusters. We also run RobustClone on this dataset; it fails to decipher the clonal architecture and predicts all mutations to be false positives, which is consistent with the result on the simulated dataset D3. The tumor phylogeny can then be efficiently obtained by using phylogeny estimation methods such as SCITE and GRMT based on the inferred 18 subclones by SCClone.

## 4 Discussion

scDNA-seq provides an unprecedented view of the genetic diversity of single cells in cancer. In this study, we introduce a novel computational method, SCClone, to cluster single cells from scDNA-seq data. It finds distinct clusters in single cells using a probability mixture model where the technical noises in scDNA-seq data are parameterized. The model parameters are efficiently estimated *via* an EM algorithm. To infer the number of underlying subclones, a score metric based on inter-cluster variance is proposed to compare models associated with different number of subclones. When compared to the state-of-the-art methods on simulated datasets, SCClone shows superior robustness against different noises in scDNA-seq data. Further evaluation results on real scDNA-seq datasets show SCClone gets consistent results with the existing methods.

As done in previous methods (Jahn et al., 2016; Zafar et al., 2017), SCClone takes an implicit assumption that genotyping errors are uniformly distributed along genomic loci, which may not hold for scDNA-seq data with severe amplification bias. In addition, SCClone does not explicitly model doublet events; thus, it may suffer from degraded performance when processing scDNA-seq data with severe contamination of doublets; we will elaborate on these issues in the future. Further improvements of SCClone can be made from multiple aspects. First, copy number information has been used to find loss-supported tumor trees (Satas et al., 2020) and can also be utilized in SCClone to yield more accurate clustering of the single cells. Second, boost in inference accuracy has been observed in joint analysis of bulk and scDNA-seq data (Malikic et al., 2019), and inclusion of bulk data is a feasible way to refine the results of SCClone. Third, information about lineage relationship between subclones is helpful to accurately estimate the genotypes of subclones, and joint inference of subclones and their lineage tree is an effective way to improve SCClone. Finally, information of genotype uncertainty may be helpful to decipher true genotypes from severely disturbed scDNA-seq data (Wu, 2020) and can be exploited as prior knowledge in SCClone to deliver more accurate results.

## Data Availability

The original contributions presented in the study are included in the article/Supplementary Material; further inquiries can be directed to the corresponding author.
